# The *Pseudomonas putida *Lon protease is involved in *N*-acyl homoserine lactone quorum sensing regulation

**DOI:** 10.1186/1471-2180-7-71

**Published:** 2007-07-26

**Authors:** Iris Bertani, Giordano Rampioni, Livia Leoni, Vittorio Venturi

**Affiliations:** 1 International Centre for Genetic Engineering and Biotechnology, Padriciano, 99, 34012, Trieste, Italy; 2 Department of Biology, University Roma Tre, Viale Marconi, 446, 00146, Rome, Italy

## Abstract

**Background:**

In *Pseudomonas putida *and *Pseduomonas aeruginosa*, the similar PpuR/RsaL/PpuI and LasR/RsaL/LasI acyl homoserine lactones (AHLs) quorum sensing (QS) systems have been shown to be under considerable regulation by other global regulators. A major regulator is the RsaL protein which strongly directly represses the transcription of the *P. putida ppuI *and *P. aeruginosa lasI *AHL synthases. In this study we screened a transposon mutant bank of *P. putida *in order to identify if any other regulators were involved in negative regulation of AHL QS.

**Results:**

In our screen we identified three Tn*5 *mutants which displayed overproduction of AHLs in *P. putida *strain WCS358. Two of the mutants had a Tn5 located in the *rsaL *gene, whereas in one mutant the transposon was located in the *lon *protease gene. Lon proteases play important roles in protein quality control via degradation of misfolded proteins. It was determined that in the *P. putida lon *mutant, AHL levels, PpuR levels and *ppuI *promoter activity all increased significantly; we therefore postulated that PpuR is a target for Lon. The Lon protease had no effect on AHL production in *P. aeruginosa*.

**Conclusion:**

The Lon protease is a negative regulator of AHL production in *P. putida *WCS358. The Lon protease has also been shown by others to influence AHL QS in *Vibrio fischeri *and *Agrobacterium tumefaciens *and can thus become an important regulator of AHL QS timing and regulation in bacteria.

## Background

Quorum sensing (QS) is a common form of gene regulation based on cell-density involving intercellular communication relying on the production and response to signaling molecules [1,2]. In Gram-negative bacteria, acyl-homoserine lactones (AHLs) are the most common signal molecules which were first described in the marine bacterium *Vibrio fischeri *as being involved in the cell-density dependent regulation of bioluminescence [1,3]. The general mechanism of AHL QS relies on two proteins belonging to the LuxI and LuxR protein families. LuxI-family proteins are the major class of AHL synthase enzymes whereas LuxR-family proteins form complexes with AHLs which are then able to bind at specific DNA promoter sequences (called *lux*-type boxes) of QS regulated genes affecting their expression.

AHL QS has become a paradigm for bacterial communication having the common scheme that AHLs are produced at a basal level at low cell densities. At high cell densities, the concentrations of AHLs surpasses a certain threshold (corresponding to the "quorum" cell density) allowing interaction with the LuxR-family protein and the system then usually undergoes positive feedback through increase of expression of the *luxI*-family gene resulting in strong sudden activation of AHL QS. This cell-density dependent response has evolved as a means to provide advantages to a community of bacteria by synchronizing group behavior. AHL QS has been studied in several Gram-negative bacteria and the physiological processes controlled by this system are diverse but are often related to virulence in pathogenic organisms [1,2,4,5].

The AHL QS systems are not always rigorously responding to cell-density as they are often integrated with other global regulatory responses and are thus influenced by other environmental factors [6-8]. Several systematic studies in *Pseudomonas aeruginosa *have shown that AHL QS is a global regulatory network controlling the expression of over 300 genes [9-11]. Recently other regulon studies in *P. aeruginosa *have demonstrated that the RpoS, VqsR and PprB global regulators are intimately interconnected with AHL QS regulons [8]. This is probably why some AHL QS systems are themselves regulated in response to various stimuli ensuring a timely control at the appropriate environmental conditions. In fact, the regulation of AHL QS has been particularly studied in *P. aeruginosa *highlighting that the *luxI*/LuxI and *luxR*/LuxR family genes/proteins are themselves extensively regulated. Positive regulation of the *lasI/R *and *rhlI/R*, the two AHL QS systems homologs of *luxI/R *present in *P. aeruginosa*, occurs via transcriptional regulators such as GacA, Vfr and PprB (reviewed by [6-8]). At present however it is not known whether any of these positive regulators are acting directly on the AHL QS genes and the precise stimuli affecting these positive regulatory responses are also not clear. Other regulators repress AHL QS in *P. aeruginosa *likely to ensure that it is not activated at low cell-densities. Reports of these negative regulators include the H-NS like protein MvaT, the *luxR*-like orphan QscR, the post-transcriptional regulator RsmA, the alternative sigma factors RpoS and RpoN, and the newly characterized RsaL repressor (reviewed by [6-8]). Of these repressors only RsaL has been shown to regulate directly the AHL QS genes; more precisely it regulates the expression of the *lasI *AHL synthase by binding to its promoter repressing transcription [12,13]. Interestingly we also reported that RsaL is a major negative regulator of AHL QS in *Pseudomonas putida *[14]. Unlike in *P. aeruginosa *however, in strain WCS358 there is only one AHL QS system, designated PpuI/R, which produces and responds to N-3-oxo-dodecanoyl homoserine lactone (C12-3-oxo-AHL); this system is highly identical to the LasI/R system of *P. aeruginosa *[13,14]. Localized in between the *ppuI/R *genes is the small *rsaL *repressor which when inactivated results in dramatic increase of ppuI expression and hence AHL production [14]. In *P. putida *RsaL repression of the AHL synthase gene appears to be much stronger that in *P. aeruginosa *[12,14].

In this study we were interested to investigate whether in *P. putida *WCS358 other negative *ppuI/R *(or PpuI/R) AHL QS regulators, either acting in concert with RsaL or independently, are present. By screening a Tn*5 *genomic mutant bank of strain WCS358 a negative regulatory mutant of AHL QS was identified and characterized to be inactivating a gene encoding for a Lon-like protease. It was demonstrated that this protease targeted PpuR thus affecting PpuR protein levels indicating that it is involved in regulating the AHL QS system.

## Results and Discussion

### Identification and characterization of AHL-overproducing mutants of *P. putida *WCS358

**Figure 1:**
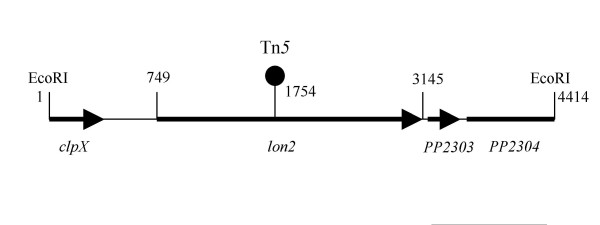
Physical map of the 4.4 kb EcoRI DNA fragment containing the *lon *protease cloned from the chromosome of *P. putida *WCS358. Position of the first and last nucleotide of the *lon *gene are shown together with the position of the Tn*5 *insertion. The ORF or part of ORFs downstream the *lon *gene display 88% and 92% similarity to the pp_2303 and pp_2304 ORFs of *P. putida *KT2440. In the upstream region of the *lon *gene, part of *clpX *ORF is localized.

In order to establish whether, besides RsaL, there were other negative regulators of the *ppuI/R *system, we screened *P. putida *WCS358 Tn*5 *genomic mutants for AHL overproduction. The genetic screen we employed here has been previously described relying on the AHL biosensor *C. violaceum *CVO26 [14,15]. *P. putida *WCS358 promotes little pigment formation in the AHL biosensor strain CVO26 because it produces very low quantities of AHLs as well as producing AHLs which have low specificity towards CviR of strain CVO26. In order to identify AHL-overproducing mutants we spread between 1000–2000 CVO26 cfu (colony forming units) and 300–500 *P. putida *WCS358 Tn*5 *mutant cfu from a Tn*5 *mutant bank on one plate and screened for strong purple loci. After screening 25,000 WCS358 Tn*5 *mutants, three mutants were scored which significantly induced violacein purple pigment production in CVO26. Two mutants were localized in the *rsaL *gene which as previously reported is an important negative regulator of *ppuI *expression and its inactivation leads to a dramatic increase of AHL production [14]. One mutant did not map in the *rsaL *gene and had the Tn*5 *inserted in an ORF of 2397 nucleotides encoding a protein of 798 amino acids (Figure 1). This ORF displayed high homology to the Lon proteases of several bacteria; over 90 % identity with proteins form several *Pseudomonas *sp. including the Lon protease of *P. aeruginosa *([16]; PA1803). The Lon protein belongs to the family of ATP-dependent proteases which is well conserved in prokaryotes and eukaryotes and has been associated with various cellular activities [17]. Lon proteases play important roles in protein quality control via degradation of misfolded proteins. Lon proteolysis can also be crucial for controlling the protein levels of regulatory proteins thus affecting programs of gene expression [17].

**Figure 2:**
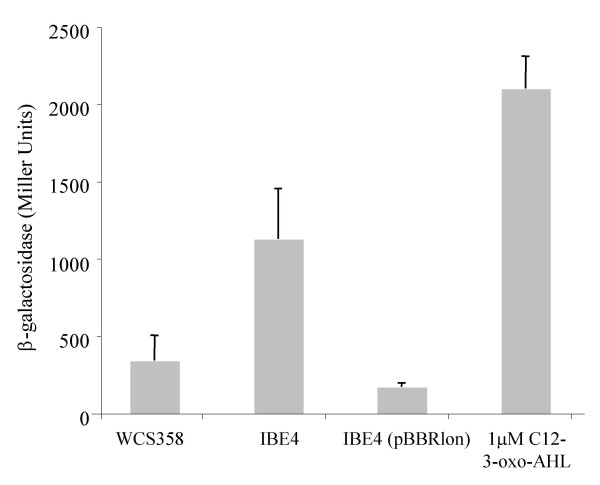
C12-3-oxo-AHL measurement produced by *P. putida *WCS358, by *lon *protease mutant derivative *P. putida *IBE4 and *lon *mutant containing a plasmid expressing the Lon protease, *P. putida *IBE4 (pBBRlon). C12-3-oxo-AHL was extracted from spent supernatants, AHL levels were measured with *P. putida *SM17 (prsaL220) with a volume of extract corresponding to an amount of 5 × 10^8 ^cfu as described in the Methods section. C12-3-oxo-AHL levels are proportional to β-galactosidase activity (Miller Units). Standard deviation bars are given on the mean value of three independent cultures. Statistical analysis was performed with *Anova *resulting in a significant main effect of the mutation with F(2,12) = 20.45 and p < .001. The same measurement was also performed using 1 μM of synthetic C12-3-oxo-AHL (obtained from P. Williams, University of Nottingham, UK).

**Figure 3:**
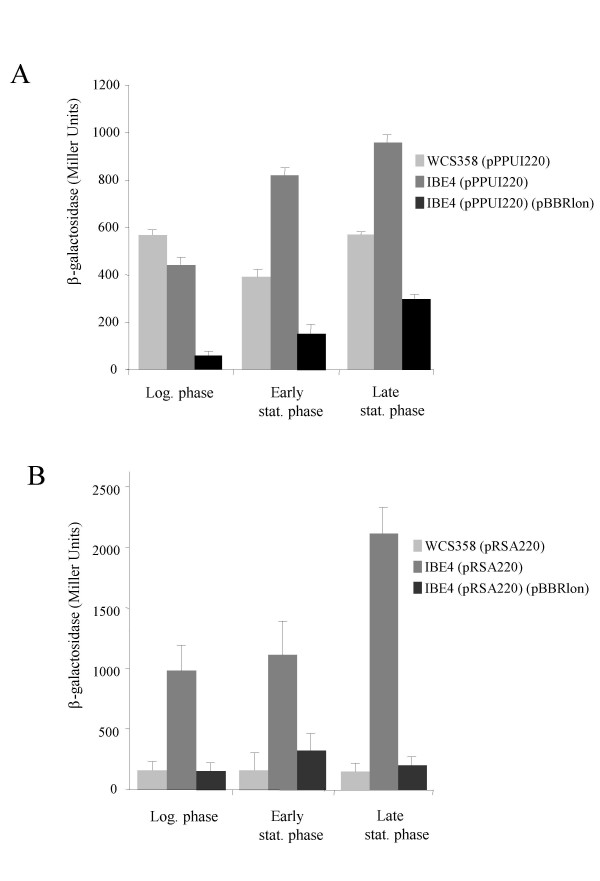
Gene promoter activities of *ppuI *and *rsaL *in *P. putida *WCS358 and *lon *mutant derivatives. A. Promoter activities of the *ppuI *C12-3-oxo-AHL synthase in *P. putida *WCS358 (pPPUI220), in the *lon *protease mutant derivative *P. putida *IBE4 (pPPUI220) and *lon *mutant containing a plasmid expressing the Lon protease, *P. putida *IBE4 (pPPUI220)(pBBRlon). The *ppuI *promoter activities are expressed in β-galactosidase activity (Miller Units) as the plasmid construct pPPUI220 contains the *ppuI *promoter transcriptionally fused to a promoterless *lacZ *gene (see text for all details). Standard deviation bars are given on the mean value of three independent experiments. Logarithmic phase (Log.) corresponds to 8 hours of growth, stationary phase (Stat.) to 16 hrs and late stationary (Late stat.) to 48 hours of growth. Statistical analysis was performed with *Anova *resulting in a significant main effect of the mutation with F(1,14) = 18.90 and p < .001. Statistical analysis including the age of the cultures results in a significant difference between the wild type and the IBE4 mutant in the Logarithmic phase of growth [F(1,14) = 28.70 p < .001]. B. Promoter activities of the *rsaL *in *P. putida *WCS358 (pRSA220), in the *lon *protease mutant derivative *P. putida *IBE4 (pRSA220) and *lon *mutant containing a plasmid expressing the Lon protease, *P. putida *IBE4 (pRSA220)(pBBRlon). The *ppuI *promoter activities are expressed in β-galactosidase activity (Miller Units) as the plasmid construct pRSA220 contains the *rsaL *promoter transcriptionally fused to a promoterless *lacZ *gene. Standard deviation bars are given on the mean value of three independent experiments. Logarithmic phase (Log.) corresponds to 8 hours of growth, stationary phase (Stat.) to 16 hrs and late stationary (Late stat.) to 48 hours of growth. Statistical analysis was performed with *Anova *resulting in a significant main effect of the mutation with F(1,18) = 126.12 and p < .000001. Statistical analysis including the age of the cultures results in a significant difference between the wild type and the IBE4 mutant in the late stationary phase of growth [F(1,18) = 18.79 p < .001].

**Figure 4:**
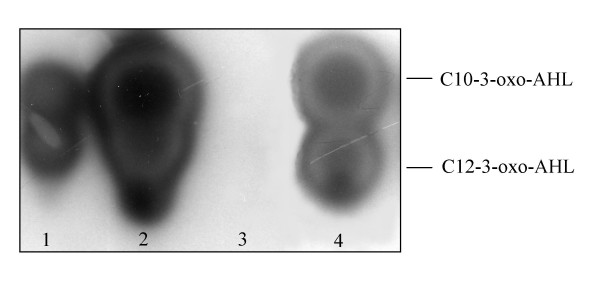
TLC analysis of C12-3-oxo-AHL produced by parent strain *P. putida *WCS358 (lane 1), by *lon *mutant derivative *P. putida *IBE4 (lane 2) and *lon *mutant derivative carrying a plasmid expressing Lon *P. putida *IBE4 (pBBRlon) (lane 3). Lane 4 contains the standards C10-3-oxo-AHL and C12-3-oxo-AHL. The AHLs were visualized with AHL-sensor strain *E. coli *(pSB1075); a volume corresponding to 5 × 10^8 ^cfu was loaded on the TLC assay.

This *lon *protease mutant identified here was designated *P. putida *IBE4 and produced three times more C12-3-oxo-AHL as determined through AHL quantification experiments as well as through TLC analysis (Figure 2 and Figure 4). This increase of AHL production was also justified by higher *ppuI *promoter activity; *ppuI *encodes for PpuI the AHL synthase enzyme (Figure 3A). As previously reported [14]*ppuI *expression does not increase with cell-density and is low in the wild-type as the QS system is strongly negatively regulated by RsaL. In fact in *P. putida *RsaL appears to be the on/off switch of the system. It not known yet what is the stimulus which leads to RsaL de-repression. It is clear however form our data that absence of Lon increases significantly *ppuI *transcription. Similarly, also the *rsaL *quorum sensing PpuR-regulated promoter displayed significantly higher activities in the *lon *protease mutant (Figure 3B). Importantly, by providing the *lon *protease in trans via plasmid pBBRlon, which carries the cloned genomic locus harbouring the *lon *protease gene expressed from its own promoter, C12-3-oxo-AHL levels, and *ppuI *and *rsaL *promoter activities were restored to wild-type levels (Figures 2, 3 and 4). From the TLC analysis it was actually observed that extra copies of *lon *gene carried in a plasmid dramatically reduced AHL production (Figure 4). It was therefore postulated that Lon could target some component(s) of the PpuI/RsaL/PpuI AHL system being therefore involved in QS regulation in *P. putida*.

### The *P. putida *Lon protease affects PpuR protein levels

**Figure 5:**
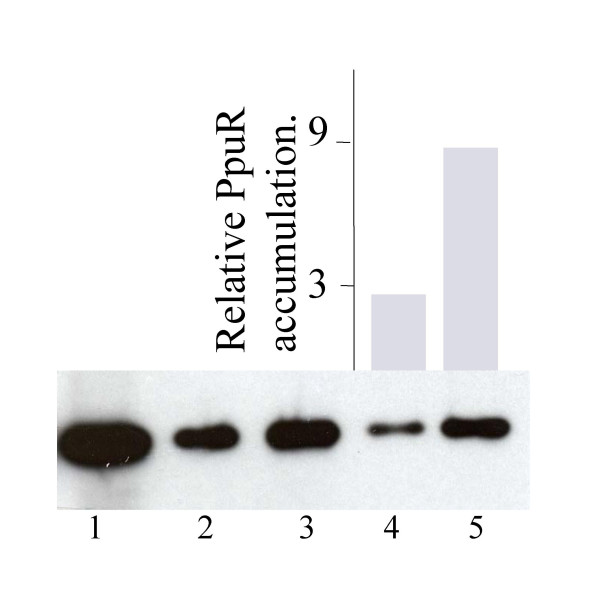
Western hybridization performed using anti-PpuR antibody on total cellular protein extracts. Total protein extracts were performed using different protein amounts of either parent strain *P. putida *WCS358 or *lon *mutant derivative *P. putida *IBE4 each carrying a plasmid expressing PpuR (pBBRppuR). Lane 1 corresponds to 10 μg of purified PpuR-6His; lane 2 and lane 3 correspond to total proteins from 2 × 10^7 ^cfu of WCS358(pBBRppuR) and IBE4(pBBRppuR) respectively; lanes 4 and 5 correspond to total proteins from 10^7 ^cfu. For lanes 4 and 5 the images were scanned using a Versadoc (Biorad) and the QuantityOne software; results of this quantification are displayed as a histogram. See text for all details.

Having established that the *lon *knock-out mutant *P. putida *IBE4 resulted in increased *ppuI *promoter activity and hence higher AHL production it was of interest to determine if the possible target of the Lon protease was the PpuR protein. The reason being that as PpuR positively regulates *ppuI *and *rsaL *expression in a positive induction loop typical of LuxI/R-type QS systems, altering PpuR levels could therefore affect AHL production through *ppuI *and *rsaL *promoter activities. In order to determine if PpuR was a target of the Lon protease we raised polyclonal antibodies against PpuR in order to visualize PpuR protein levels. We therefore examined levels of PpuR in 16-h-old stationary phase cultures of *P. putida *WCS358 and *lon *protease knock-out mutant *P. putida *IBE4 using anti-PpuR antibodies. The levels of PpuR protein were found to be very low and hardly detectable in strain WCS358; this also reflected the very low *ppuR *promoter activities which were previously observed [14]. In order to be able to better visualize PpuR levels we introduced in both the wild type and mutant derivative IBE4, the plasmid pBBRppuR which carries the *ppuR *gene expressed from the *lac *promoter (Figure 1; Table 1). As depicted in Figure 5, PpuR levels in *P. putida *IBE4 were detected as being significantly higher than those in the wild type parent strain indicating that absence of the Lon protease resulted in larger amounts of PpuR present in the cell. This experiment was reproducible being repeated three times obtaining similar results also loading different amounts of total proteins (data not shown). We verified that *ppuR *promoter activity in the *lon *mutant was not affected (data not shown) in order to exclude that Lon might have been acting on other proteins components affecting *ppuR *transcription. This provides indirect evidence that the Lon protease targets PpuR thus being able to regulate quorum sensing in *P. putida*. Other LuxR-family quorum sensing regulators have also been shown to be targeted by the Lon protease. The TraR protein of *Agrobacterium tumefaciens *was shown to be susceptible to Lon proteolysis when free of AHL-ligand [18]. Similarly, LuxR of *Vibrio fischeri *complexes in vivo with Lon degrading it; in a Lon mutant it was also observed that LuxR accumulates at higher levels [19]. It therefore appears that many members of this family could be targets of the Lon protease in order to control their levels; Lon can consequently be regarded as a regulator of quorum sensing ensuring the correct timing of the response. At present we do not know whether the *lon*/Lon protease is itself regulated thus adding a further element(s) of control of QS in *P. putida*. Regulation of QS in *Pseudomonas *has been shown to be very complex and intricate involving a myriad of global regulators; this indicates that timing and response of the QS system is important and the Lon protease must now be added to this regulatory circuit. Other proteases have also been implicated in targeting members of the LuxR-protein family [18]. Lon has also been reported to affect the accumulation of several other types of transcriptional regulators in bacteria thus affecting transcription of important global regulatory systems [17,20-22]. For example, Lon influences the regulation of antibiotic production in *Pseudomonas fluorescens *Pf-5 through what is postulated to be degradation of of a positive regulator [21]. In *P. aeruginosa*, the Lon protease was found to be important for biofilm formation and motility [20] and pathogenicity and type III secretion in *Pseudomonas syringae *are regulated by Lon via an effect on the stability of transcriptional regulators [19].

**Table 1:** *Pseudomonas *strains, plasmids and oligonucleotides used.

**STRAINS**		
*P. putida *WCS358	*Pseudomonas putida*, wt	[33]
*P. putida *IBE4	*lon1754*::Tn*5 *of WCS358, Km^r^	This study
*P. aeruginosa *18577	*PA1803lon437*::ISlacz/hah of PAO1, Tc^r^	[34]
		
**PLASMIDS**		
pMOSBlue	Cloning vector, Amp^r^	Amersham-Pharmacia
pBluescriptKS	Cloning vector, Amp^r^	Strategene
pRK2013	Km^r ^Tra+ Mob+, ColE1 replicon	[28]
pET28b	Expression vector, km^r^	Novagen
pBBRmcs5	Broad-host-range vector, Gm^r^	[35]
pSCHIA5	pBluescriptKS carrying a 4.5 Kb BamHI/StuI fragment from IBE4 harboring some WCS358 and a fragment of Tn*5*, Amp^r ^Km^r^	This study
pB1A	pBluescriptKS carrying a 4.5 Kb EcoRI fragment from WCS358, Amp^r^	This study
pBBRlon	pBBRmcs5 carrying a 4.5 Kb EcoRI fragment from WCS358, Gen^r^	This study
pPPUR3586H	pET28b derivative carrying *ppuR *of *P. putida *WCS358	This study
pBBRppuR	pBBRmcs5 carrying the ppuR gene amplified from pPuR3586H with primers P0FW and P0RV	This study
pPPUI220	pMP220 promoter probe vector carrying the *ppuI *promoter region, Tc^r^	[14]
pRSA220	pMP220 promoter probe vector carrying the *rsaL *promoter region, Tc^r^	[14]
		
**OLIGONUCLEOTIDES**		
P0FW	5'-GGGTACCAATAATTTTGTTTAACTTTA-3'	[12]
P0RV	5'-GGGATCCATTGCTCAGCGGTGGCAGC-3'	[12]
FW119	5'-CATGCCATGGCCCTACTGGTAATGG-3'	This study
RV122	5'-CCCAAGCTTGGGCGTGATCGATTTTTGC-3'	This study
Tn5ext	5'-GAACGTTACCATGTTAGGAGGTC	[36]

### The *P. aeruginosa *Lon protease does not affect C12-3-oxo-AHL production

**Figure 6:**
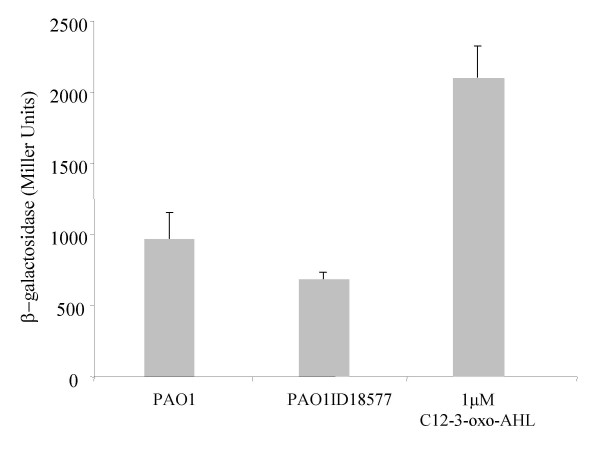
C12-3-oxo-AHL measurement produced by of *P. aeruginosa *PAO1 and by *lon *protease mutant derivative *P. aeruginosa *18577. C12-3-oxo-AHL was extracted from spent supernatants, AHL levels were measured with *P. putida *SM17 (prsaL220) with a volume of extract corresponding to an amount of 5 × 10^8 ^cfu as described in the Methods section. C12-3-oxo-AHL levels is proportional to β-galactosidase activity (Miller Units). Standard deviation bars are given on the mean value of three independent cultures. Differences between PAO1 and PAO1 ID18577, analysed using T-test for independent groups were not significant (t = 2.2; p = .06; df = 6;t(6) = 2.2). The same measurement was also performed using 1 μM of synthetic C12-3-oxo-AHL (obtained from P. Williams, University of Nottingham, UK).

The Lon protease of *P. putida *WCS358 displayed almost 90% amino acid identity over the entire length of the protein with the Lon protease of *P. aeruginosa *(PA1803; [16]). In addition, the PpuI/RsaL/PpuR AHL QS system of strain WCS358 is highly similar to the LasI/RsaL/LasR of *P. aeruginosa*; they both produce and respond to C12-3-oxo-AHL and the three proteins are highly identical [12-14]. Since the two species are very close phylogenetically and the two AHL QS systems are orthologs with PpuR and LasR being highly identical, it was of interest to determine if also in *P. aeruginosa *the Lon protease played a role in AHL QS regulation. We therefore determined C12-3-oxo-AHL levels in wild-type *P. aeruginosa *PAO1 and *lon *protease mutant *P. aeruginosa *18577. Differently to what occurs in *P. putida *WCS358, the *lon *protease knock-out mutant of *P. aeruginosa *produced comparable amounts of C12-3-oxo-AHL to wild-type parent strain PAO1 (Figure 6). This indicated that unlike what occurred in *P. putida *WCS358, in *P. aeruginosa*, Lon most probably does not target proteins of the LasI/RsaL/LasR AHL QS system. It was observed however that in the *P. aeruginosa *PAO1 genome, an ORF of 795 amino acids (PA0779) displayed 40% identity with the Lon protease is present thus it cannot be excluded that other similar proteases are present which can target protein(s) of AHL QS systems.

## Conclusion

In this study we determined that the Lon protease is a negative regulator of AHL production in *P. putida *WCS358. AHL production and response in WCS358 occurs via the PpuR/RsaL/PpuI AHL QS system; this system is highly homologous to the LasR/RsaL/LasI system of *P. aeruginosa*. It was observed that in a Lon mutant, C12-3-oxo-AHL levels, PpuR levels and *ppuI *promoter activity all increase significantly; we therefore postulated that PpuR is a target for Lon. Unlike what occurs in *P. putida *WCS358, in *P. aeruginosa *Lon has no effect on AHL production. It was however observed that *P. aeruginosa *possesses in its genome another ORF with very similar features to Lon thus it cannot be excluded that other proteases could be involved in AHL QS regulation. As two other LuxR family regulators have been shown to be targeted by Lon, it is concluded that proteases could play an important role in AHL QS timing and regulation.

## Methods

### Bacterial strains, plasmids and media

The bacterial strains and plasmids used in this study are listed in Table 1. *Pseudomonas putida *WCS358 is a plant growth-promoting strain isolated from the rhizosphere of potato roots.*Chromobacterium violaceum *CVO26 is a double mini-Tn*5 *mutant derived from ATCC31532, this mutant is non-pigmented and production of the purple pigment can be induced by providing exogenous AHL inducer molecules [23]. *Escherichia coli *DH5α [24]and *C. violaceum *CV026 were grown in LB medium [25] at 37°C and 30°C respectively. *Pseudomonas *was grown in LB medium or M9 minimal medium [25] at 30°C. The following antibiotic concentrations were used: ampicillin (Amp) 100 μg/ml; kanamycin (Km) 100 μg/ml; nalidixic acid (Nx) 25 μg/ml; tetracycline (Tc) 10 μg/ml (*E. coli*), 40 μg/ml (*Pseudomonas*); chloramphenicol (Cm) 25 μg/ml (*E. coli*), 250 μg/ml (*Pseudomonas*); gentamycin (Gm) 10 μg/ml (*E. coli*), 40 μg/ml (*Pseudomonas*).

### Recombinant DNA techniques

DNA manipulation as digestion with restriction enzymes, agarose gel electrophoresis, purification of DNA fragments, ligations with T4 ligase, end-filling with Klenow enzyme, hybridization, radioactive labeling by random priming and transformation of *E. coli*, were performed as described previously [25]. Southern hybridizations were performed using N+Hybond membrane (Amersham Biosciences); plasmids were purified using the Jet star colums (Genomed, GmbH, Germany) or with the alkaline lysis method [26]; total DNA from *Pseudomonas *was isolated by Sarkosyl/Pronase lysis as described previously [27]. Triparental matings between *E. coli *and *P. putida *were carried out with the helper strain *E. coli *DH5α (pRK2013) [28].

### Isolation of an AHL over-expressing genomic mutant and cloning of a lon-like protease of *P. putida *WCS358

In order to identify *P. putida *WCS358 mutants that overproduce AHL, a Tn*5 *genomic mutant library was screened against the AHL biosensor strain *C. violaceum *CVO26. *P. putida *WCS358 induced only slightly pigmentation when streaked in close proximity to strain *C. violaceum *CVO26. This screening for AHL over-producers used here was previously employed to isolate *rsaL *mutants on *P. putida *WCS358 [14]. 25,000 Tn*5 *genomic mutants of *P. putida *WCS358 were screened against CVO26 for AHL overproducer mutants as previously described [14,15]. This led to the identification of one Tn*5 *mutant (designated *P. putida *IBE4), of which the Tn*5 *was not located in the *rsaL *gene and could induce strongly pigmentation of CVO26. The position of the Tn*5 *was determined after cloning from the mutant IBE4 chromosome a 4.2 kb BamHI-StuI fragment in pBluescript KS yielding pSCHIA5, which contained part of Tn5 (including the Km resistance gene) and part of adjacent WCS358 DNA. The adjacent DNA to the Tn5 was sequenced using as primer the oligonucleotide sequence Tn5ext (see Table 1) which was designed against the border of the Tn5 DNA sequence. The 4.2 kb BamHI-StuI fragment was then used as a probe to clone a 4.5 kb EcoRI from the chromosome of parent strain WCS358 in pBluescript KS yielding pB1A. This latter EcoRI fragment was sequenced (sequencing service, CRIBI, University of Padua, Padua, I) revealing that the Tn5 was positioned within an ORF of 2397 nucleotides encoding for a putative Lon-like protease of 799 amino acids (Figure 1).

### Reporter gene fusion assay

β-galactosidase activities were determined during growth in LB medium essentially as described by Miller *et al*., [29] with the modifications of Stachel *et al*. [30]. All experiments were performed in triplicate and the mean value is given. The growth curves of all mutants were comparable to the one obtained for the parent strain.

### Purification and quantification of C12-3-oxo-AHL

The purification, detection and characterization of AHLs were performed as previously described [23,31]. *Pseudomonas *strains were grown O/N in M9 minimal medium supplemented with citric acid and the OD_600 _was measured. The spent culture supernatants were extracted two times with the same culture volume of ethyl acetate-0.1% acetic acid. The extracts were then dried and resuspended in ethyl acetate with an amount which resulted in 1 μl of final extract corresponding to 2 × 10^7 ^cells of the original culture. The quantity of C12-3-oxo-AHL in the extracts was determined using the specific 3-oxo-C12-AHL sensor *P. putida *SM17 (prsal220) as previously described [13]. Briefly, *P. putida *SM17 is a double mutant of *ppuI *and *rsaL *genes consequently it does not produce the RsaL repressor and 3-oxo-C12-AHL. Adding exogenous 3-oxo-C12-AHL is quantified through β-galactosidase activity by using stain SM17 harboring (prsal220); this plasmid contains the PpuR-3-oxo-C12-AHL regulated *rsaL *promoter fused to promoterless *lacZ *gene. Overnight cultures of SM17(prsal220) were diluted in 10 ml of LB medium to an A_660 _of 0.1; the AHL extract to be quantified was then added and after 6 hours of growth β-galactosidase activity was determined. This 3-oxo-C12-AHL bacterial sensor has a linear dose response between 0.1 μM to 5 μM of 3-oxo-C12-AHL. Synthetic 3-oxo-C12-AHL was used as standard molecules (obtained from P. Williams, University of Nottingham, UK). The detection of the AHLs on the TLC plate was obtained overlaying the TLC plate with a thin layer of LB top-agar seeded with *E. coli *(pSB1075) as previously described [14,31,32].

### PpuR antibodies and protein anaylsis

Antibodies against PpuR of *P. putida *were generated by injecting purified protein into rabbits. *P. putida *PpuR was purified as PpuR-His6 using expression plasmid pET28b (Novagen); *ppuR *was cloned as a 729 PCR fragment originated from primers FW121 and FW122 using *P. putida *WC358 genomic DNA as template and was cloned as a NcoI-HindIII fragment in pET28b yielding pPPUR3586H. This latter plasmid was introduced in *E. coli *BL21(DE3)pLys (Novagen) which then resulted in the isopropyl-β-D-thiogalactopyranoside-induced over-expression of PpuR tagged with six histidines at the C- terminus. The purification of PpuR-His6 was then carried out by Ni ^2+ ^affinity under denaturing conditions according to the standard procedure suggested by the column manufacturer (Sigma-Aldrich, St. Louis, Mo.).

Proteins were transferred onto PVDF membrane (Immobilon-P; Millipore) using a tank system according to the manufacturer's instruction. The membrane was subjected to Western analysis using anti-PpuR polyclonal antibodies raised in rabbits and polyclonal goat anti rabbit immunoglobulins HRP (DakoCytomation, Glostrup, DK) and developed using Immun-Star HRP Substrate kit (BioRad laboratories, Hercules CA, USA). No significant cross-reaction of the polyclonal antibody against other *P. putida *WCS358 proteins was observed in this study.

### DNA sequencing and nucleotide sequence accession numbers

All DNA sequences were performed at the CRIBI center (University of Padua, Italy) and the nucleotide sequence of the 4.414 EcoRI fragment has been deposited in GenBank/EMBL/DDBJ under the following accession number AM690373.

## Authors' contributions

IB performed the screening of the Tn5 mutant bank, lon gene cloning and sequencing, AHL quantifications, gene promoter assays and western analysis. GR cloned *ppuR *in the expression vector and purified PpuR for raising antibodies in rabbits. LL was involved in supervision of GR work and discussions and planning with VV. VV participated in experimental design and data analysis, coordinated the study and drafted the manuscript. All authors read and approved the manuscript.
